# An Evaluation of Two Different Broiler Catching Methods

**DOI:** 10.3390/ani8080141

**Published:** 2018-08-15

**Authors:** Käthe Elise Kittelsen, Erik Georg Granquist, Agnete Lien Aunsmo, Randi Oppermann Moe, Elisiv Tolo

**Affiliations:** 1 Animalia, the Norwegian Meat and Poultry Research Centre, N-0585 Oslo, Norway; elisiv.tolo@animalia.no; 2 NMBU—the Norwegian University of Life Sciences, Faculty of Veterinary Medicine, N-0454 Oslo, Norway; erikgeorg.granquist@nmbu.no (E.G.G.); Randi.moe@nmbu.no (R.O.M.); 3 Norsk Kylling AS, N-7290 Støren, Norway; agnete.lien.aunsmo@norsk-kylling.no

**Keywords:** animal welfare, broiler, catching, pre-slaughter chain, poultry, wing fractures

## Abstract

**Simple Summary:**

Catching is the process that transfers birds from the poultry house to the transport modules. The catching process and its associated handling may lead to stress, injuries, mortality and reduced welfare for the animals. The aim of this pilot study was to investigate the effect of two manual broiler catching methods. Broilers were either caught by both legs and carried inverted to the drawers or caught under the abdomen and carried in an upright position. Effects of catching method on crating time, number of animals in the drawers, wing and leg fractures, animals on their back in the drawers and broilers dead-on-arrival were investigated. The results showed that the abdominal and upright method was faster and gave a lower and more consistent number of birds per drawer. In addition, this method tended towards fewer wing fractures. No broken legs, birds on their back in the drawers or broilers dead-on-arrival were observed in the study. Catching is a critical phase in the pre-slaughter chain, and this study shows that the catching and carrying method affects broiler welfare.

**Abstract:**

Catching is the first step in the pre-slaughter chain for broiler chickens. The process may be detrimental for animal welfare due to the associated handling. The aim of this pilot study was to compare two different methods to manually catch broilers: Catching the broilers by two legs and carrying them inverted (LEGS) or catching the broilers under the abdomen and carrying them in an upright position (UPRIGHT). Wing and leg fractures upon arrival at the abattoir, animal density in the drawers, birds on their back, broilers dead-on-arrival and time to fill the transport modules were investigated. The results showed that mean crating time was shorter in the UPRIGHT method (*p* = 0.007). There was a tendency for more wing fractures in broilers caught by the LEGS (*p* = 0.06). The animal density in the drawers was lower and with a smaller range in the UPRIGHT method (*p* = 0.022). The results indicate that catching the broilers under the abdomen in an upright position may improve broiler welfare in terms of fewer wing fractures, more consistent stocking density in drawers and potentially reduced loading time.

## 1. Introduction

Catching is the first step in the pre-slaughter chain of broiler chickens [1]. In this process, the broilers are transferred from the floor of the broiler house into transport modules, which are then loaded onto vehicles for transport to the abattoir.

The catching process may be performed mechanically or manually [2]. Under commercial manual catching conditions, the broilers are commonly caught and carried by one leg, with three to five broilers in one hand and one or two in the other hand [3,4]. The broilers are carried inverted to the transport module, which is usually a container with several drawers where the animals are crated. The catching and crating process may cause stress [1,2,5,6], injuries, and mortality [7,8,9] and hence, compromise the welfare of the broiler chickens. The Humane Slaughter Association and EU’s “Animal Transport Guides” (Consortium of the Animal Transport Guides Project) recommend catching birds individually with a grip over the wings, in an upright position [3,10]. The same catching method is recommended in Brazil due to a reduced risk of injuries for the birds [11]. Catching and carrying in an upright position can reduce handling stress, measured as corticosteroid concentration [12,13]. Furthermore, handling in an inverted position leads to prolonged tonic immobility, which is a commonly used measure of fear [14]. If the birds are to be caught and carried inverted, several organizations recommend that the best practice is to catch the birds by both legs [3,15]. Approximately four billion broiler chickens are kept for meat production in the European Union [16]. The number of birds implies that injuries and poor welfare even in a small portion of the poultry population affects a large number of animals [17]. Hence, reducing stress and injuries during catching and carrying is of utmost importance.

Knowledge, skills and training of personnel involved in handling animals are fundamental in improving the welfare of commercial livestock [18]. However, there is little scientific literature concerning different manual catching methods of poultry. It is, therefore, necessary to improve scientific knowledge regarding how different manual catching methods affect broiler welfare. This knowledge can be used by the industry to ensure that catchers are trained according to best practice. The aim of this study was, therefore, to assess the effects of two different manual catching methods; catching by two legs or catching under the abdomen in an upright position. Since catching may affect animal welfare, as well as put a strain on the workers, logistics and economy, the following indicators were recorded: Crating time of individual transport modules, the number of birds in each drawer, wing and leg fractures, birds on their back in the drawers observed in lairage, and broilers dead-on-arrival (DOA).

## 2. Materials and Methods

**Figure 1:**
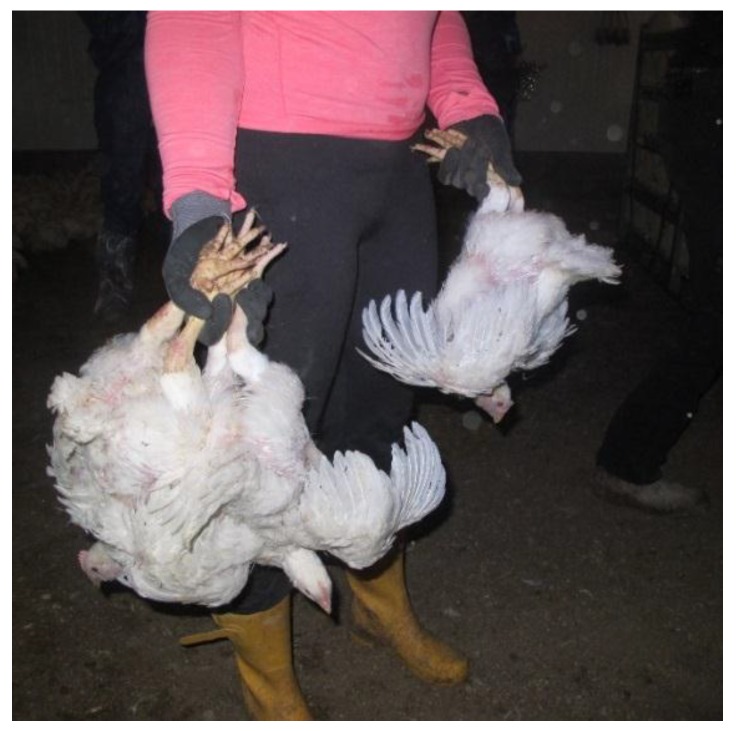
Catching and carrying broilers by a grip in both legs.

The pilot study was designed to evaluate two different methods of manual broiler catching. LEGS: The broilers were caught by two legs and carried upside-down (Figure 1). Usually, this method allowed two or three broilers in one hand and one in the other hand (range: Two to four birds in one hand, one to two in the other).

**Figure 2:**
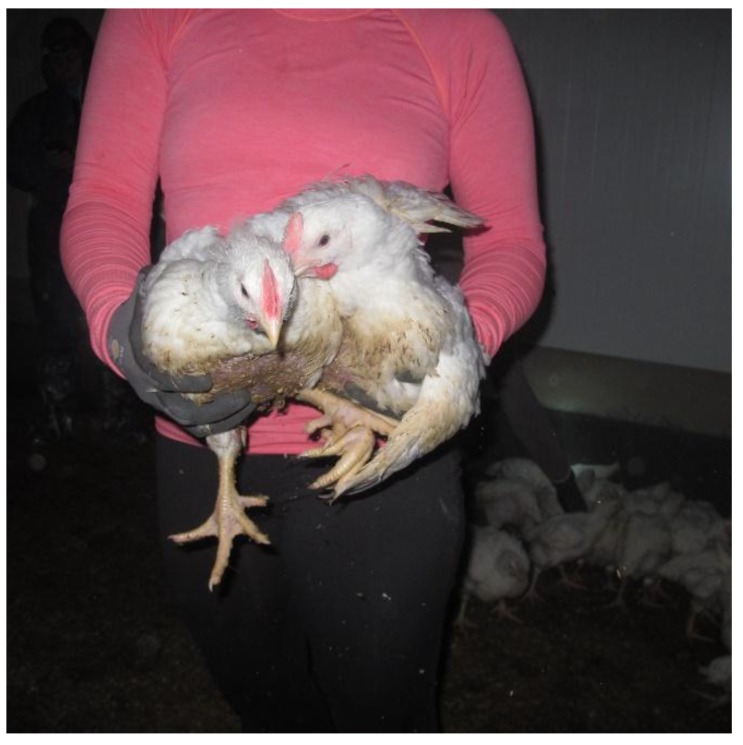
Catching under the abdomen and carrying the broilers upright.

UPRIGHT: The broilers were caught under the abdomen (Figure 2) and carried in an upright position to the transport modules. Mostly two broilers were caught and carried in each catch by the upright method (range: One to two birds per catch; a catch refers to each time the catchers pick up birds and place them in the drawer).

The study was carried out under commercial conditions in one major broiler producing region in Norway (Trøndelag). Two flocks from two different farms were enrolled. The two flocks were of different hybrids; one flock was a conventional fast-growing type (Ross 308), flock size 30,000 broilers, slaughtered at the age of 33 days and 1328 g (mean carcass weight for the flock, estimated live weight 2000 g) and the other flock was a slower growing hybrid (Hubbard JA 787), flock size 16,800 broilers, slaughtered at the age of 44 days and 1539 g (mean carcass weight for the flock, estimated live weight 2300 g). Both flocks were mixed sex and fed ad-libitum. Both broiler houses were from the same manufacturer, had the same size and were equipped with the same ventilation system.

The study sample consisted of 3951 broilers from both houses in total; 2010 caught by LEGS (1031 broilers from the conventional hybrid and 996 broilers from the slower growing hybrid) and 1941 by UPRIGHT (969 broilers from the conventional hybrid and 955 broilers from the slower growing hybrid). This implies that the majority of the broilers in the two flocks were not included in the study. Both catching methods were evaluated in both flocks.

The sampling was carried out in dimmed lighting at the start of the catching process. Only the first eight transport modules in each house were included in the study; the first four modules with LEGS, then the next four modules with UPRIGHT. The birds were caught randomly for each method. The two broiler flocks were caught two subsequent nights by the same catching team, consisting of the same four professional catchers. The team consisted of two women and two men; all had catching as their primary occupation. They had no standard catching method, sometimes by one leg, sometimes by two legs and sometimes upright by abdomen. For this study, they were trained to catch both by LEGS and by UPRIGHT methods. The training consisted of theoretical instructions along with practical demonstrations. For LEGS, the only instruction was to catch by both legs for each bird. For UPRIGHT, the only instruction was to catch under the abdomen and carry the broilers in an upright position. The instruction did not regulate the maximum number of birds allowed per catch. All catchers performed both methods in this study.

Both flocks were caught late night/early morning. The size of the transport modules was identical for both hybrids and methods (Stork^®^, 2.43 × 1.30 m [length × width], eight open-topped drawers in a module, a planned minimum of 200 cm^2^ per kg live bird). The transport modules were marked according to catching the method, and the eight modules were loaded on the same vehicle. The time to fill each transport module with birds was recorded. The modules were loaded in and out of the broiler house with a forklift. The truck driver placed all modules as close as possible to the broilers as per normal industry practice. This process was consistent for both methods and both flocks.

The journey time from farm to abattoir was 30 min for the conventional flock and 10 min for the slower growing flock. Immediately after the transport modules arrived at the abattoir, the broilers were manually lifted out of the modules and investigated in lairage for wing and leg fractures, birds on their back in the drawers and DOA. The birds were assessed by two of the researchers at the same time. Injured birds were immediately stunned with blunt trauma to the head, followed by cervical dislocation. The wing fracture criteria were: open or closed fractures, dislocated wings and detachment of the epiphyseal plates with visible bleeding around the elbow joints [9]. In addition, the number of animals per drawer was recorded. These investigations were performed in lairage and not on the farm, due to better lighting and protection against low temperatures at the abattoir. The assessors of the birds in lairage were aware of the catching method.

Data were entered into an Excel spreadsheet and transferred to STATA 14.2 (College Station, TX, USA). All observations were inspected for deviations and missing data before the analyses. The distribution of the continuous variables (crating time and stocking density) were inspected visually by histograms and by summary statistics. Both variables were approximately normally distributed. Simple linear regression was used to compare means of catching and crating time, the number of birds per cage, DOA and fractures by hybrid and catching method. An interaction term was included in the regression analyses to evaluate whether the univariable relationships between stocking density and hybrid, as well as catching time and hybrid were dependent on the simultaneous effects of hybrid and catching method. Residuals were predicted and visualized by histograms. Since wing fractures were either present or absent in each module, this variable was treated as categorical. Thus, for wing fractures, simple logistic regression was used to study the effect of birds per cage, hybrid, crating time, and catching method. Hybrid was included as a covariate in the study of effects of birds per cage and crating time on wing fractures. Residuals (linear regression) were predicted and displayed on normal quantile plots.

## 3. Results

For a summary of the main results, see Table 1. For a comparison of the two methods, broken down to flock, see Table 2.

### 3.1. Crating Time

Overall, the mean time to fill the transport module was 241 s (range 161–340; SEM 3.84). There was a significant difference in time to fill the transport modules between the two hybrids. For the conventional fast-growing hybrid, the mean time to fill was 219 s (range 161–270; SD 34.03) and 264 s (range 219–340; SD 40.12) for the slower growing hybrid (*p* = 0.001). The difference in crating time between the two hybrids when the interaction between hybrid and catching method was included was significant (*p* = 0.048). Mean crating time was significantly shorter for the UPRIGHT method; the mean time to fill a transport module by LEGS was 252 s (range 188–340; SD 41.48), versus 231 s (range 161–313; SD 43.28) for UPRIGHT (*p* = 0.007).

### 3.2. Number of Birds Per Drawer

The mean number of birds in the drawers was 30.87 (range 25–36; SD 1.90). For the conventional fast-growing hybrid, the average was 31.67 birds (range 27–36; SD 1.80) (space allowance: 220 cm^2^/kg estimated live weight), and for the slower-growing hybrid, it was 30.06 birds (range 25–35; SD 1.64) (space allowance: 197 cm^2^/kg estimated live weight). The difference in the number of hybrid animals per drawer was significant (*p* = 0.001). The interaction between hybrid and catching method was not significant (*p* = 0.273). The average number of birds in each drawer was 31.25 for LEGS (range 25–36; SD 2.10) (space allowance, regardless of hybrid: 204 cm^2^/kg, estimated live weight), and 30.48 birds for UPRIGHT (range 26–33; SD 1.59) (space allowance, regardless of hybrid: 211 cm^2^/kg, estimated live weight) (*p* = 0.022).

### 3.3. Wing Fractures

Wing fractures were not a common finding in this study; in total eight wing fractures were observed upon investigation in lairage. Two fractures were observed in the conventional fast-growing hybrid and six fractures in the slower growing hybrid (*p* = 0.164). Seven wing fractures were observed in broilers caught by LEGS, one fracture for broilers caught by UPRIGHT. There was a tendency for higher odds (OR = 7.74) of wing fracture for LEGS versus UPRIGHT (*p* = 0.06).

### 3.4. Other Welfare Indicators

Upon examination in lairage, none of the broilers included in this pilot study were dead-on-arrival or on their back in the drawers. Likewise, no leg fractures were observed.

## 4. Discussion

Despite limitations in the number of animals enrolled in the study, the catching method was observed to affect several of the parameters recorded. Catching the broilers under the abdomen in an upright position was a faster method, gave slightly fewer wing fractures and gave a more consistent animal density in the drawers, compared to catching broilers by two legs and carrying them inverted to the drawers. A comparison with one-leg catching was not performed since this method is prohibited in Norway [19].

There was a tendency for fewer wing fractures observed in broilers caught upright under the abdomen. An overall low prevalence of wing fractures was recorded (0.20%). Since the wings were examined for fractures in the lairage, a distinction between fractures due to catching and due to transportation could not be made. However, Jacobs et al. [7] investigated wing fractures post catching and post lairage. They found 1.88% wing fractures after catching and 1.90% in lairage, indicating that the fractures are typically attributable to catching and not to transportation. Available literature on wing fractures registered prior to processing is sparse. Kittelsen et al. investigated wing fractures prior to stunning and found 0.8% wing fractures in the observed broilers [9], a slightly higher number than observed in the current study. Langkabel et al. found no significant differences in the number of wing fractures between one-leg and two-leg catching [4], however, it was reported that the animals appeared to be more restless and performed more wing flapping during two-leg catching. In the current study, no one-leg catching was investigated, but catching by two legs gave more wing flapping than catching upright under the abdomen (anecdotal observation). The results from both studies indicate that the activity of the broilers during carrying did not affect the prevalence of wing fractures. It can be hypothesized that the actual crating process represents a larger risk for wing fractures than the carrying. This may explain the tendency for fewer fractures in broilers caught upright under the abdomen, since this method had more controlled placements of the birds in the drawers. There is a possibility that the catcher’s behavior and handling of the birds were subject to observer effect. However, the catchers did not know which indicators would be assessed. The observation may have affected all handling equally, but it does not explain the differences between the catching methods. 

Crating time was included in the study since the total time taken to catch an entire flock is important for broiler welfare, as well as the logistics and economy of the industry. Prolonged catching will influence time without feed and water, and therefore cause stress to the birds [20,21]. Crating time was directly and simultaneously influenced by both the hybrid and the catching method. Catching of the commercial fast-growing hybrid was quicker compared to the slower growing hybrid, regardless of method. This may be due to the weight of the slow-growing hybrid. The results also show that catching the broilers UPRIGHT, either one or two birds at a time, was faster than getting a grip of both legs of the animals, even though more birds could be collected in one catch with the two-leg method. In accordance with Langkabel et al. [4], the catchers were observed to fumble more to collect both legs than when they lifted one or two birds upright under the abdomen (anecdotal observation). This study did not consider how the level of practice and experience that the catchers had prior to the study may have influenced the crating time. The catchers perceived the abdominal catching to be most exhausting, since this method allowed fewer birds per catch and therefore more squats. The method’s physical load on the catchers may affect crating time negatively if this method was performed in the entire flock, since prolonged depopulation time may increase stress and time without feed and water. However, it must be noted that too rapid handling also may be detrimental to bird welfare, as this may increase the risk of injuries, and as such, rapid handling by itself is not a goal. Few broilers were included in this study, compared with a commercial catching of larger flocks. Therefore, further studies are needed to assess the effect of the catching method on the crating time.

The number of broilers per drawer was higher when the birds were caught by LEGS, compared to UPRIGHT. In addition, the range in the number of birds per drawer was higher, making the crating density inconsistent with LEGS. The animal density is an important parameter during transport: If the density is too high, it gets more difficult for the birds to tackle high or low on-board temperatures and dead-on-arrivals may be the outcome [22,23,24]. The reason for this is that the mechanisms broilers use for cooling are less effective at higher densities [25]. Therefore, it is important to obtain the optimal density in each drawer. The reason for the significant difference in animal density between the two catching methods may be uneven “catches” and a larger range in the number of animals per catch. Especially with LEGS, the number of birds per catch varied between catches and between the catching personell. This made it difficult to keep track of the number of birds per drawer, especially since two catchers filled one drawer at the same time. The problem with crating density will likely be solved with more experience or a catcher designated to count the broilers.

In this pilot study, the catching method was applied randomly. However, due to the limited size of the study, LEGS was performed prior to UPRIGHT in both flocks. This may have affected our results since the catchers could have been more tired when starting abdominal catching. However, only a small proportion of the flocks were a part of this study, and the depopulation started with these two methods. Approximately 1000 broilers were caught by LEGS, followed by 1000 broilers by UPRIGHT in each flock. This equals approximately 3.33% and 5.95% of the flocks, respectively, which is only a small proportion of the animals that the catchers are used to handling each night. The impact of the catching method used when observers left the broiler house could have been used as a control, but this was not possible since the recording of wing fractures in lairage were very time-consuming. Including additional registration on controls would be negative for animal welfare in this study, as it would prolong the total crating time, and the time without feed and water for the entire flocks. In this study, the flocks were categorized as different hybrids. This may be misleading as there were several other factors that may have affected the results, e.g., live weight, management. This should be further investigated in future studies.

In previous studies on catching, bruising is included as a welfare indicator. In the current study, all registrations were observed in lairage, prior to the slaughter process. Bruising can only be observed after defeathering and was therefore not included as an indicator in this study. Several questions need to be addressed in a follow-up study with a larger sample size; these include the effect of crating time if the methods are applied in large flocks and more welfare indicators (e.g., stress evaluation and bruising).

## 5. Conclusions

Catching is a critical phase in the pre-slaughter chain, and this study shows that the catching and carrying method affects broiler welfare. One major finding is that there is a strong tendency towards more wing fractures when the birds are caught by two legs. In addition, the time to fill a transport module was less with the UPRIGHT method and resulted in a lower and more consistent animal density in the drawers compared to catching by two legs. Clearly, due to the small sample size, further studies are needed to form the basis for improved and more animal-friendly catching routines. It must be noted that both methods are likely to slow down the catch rate compared to one leg capture and this may have economic implications for the industry due to increased personnel costs. The consequence of this is unknown. However, catching by one leg is prohibited and alternative catching methods need to be implemented.

## Figures and Tables

**Table 1:** Outcome measures of upright and leg catching methods, trialled in one conventional and one slow-growing flock of broilers.

Indicators	Mean	Conventional Hybrid ^1^, N = 2000	Slower Growing Hybrid ^2^, N = 1951	*p*-Value, Conventional vs. Slow-Growing Hybrid	LEGS, N = 2010	UPRIGHT, N = 1941	*p*-Value, LEGS vs. UPRIGHT
Crating time ^3^	241	219	264	0.001	252	231	0.007
Number of birds per drawer	30.87	31.67	30.06	0.001	31.25	30.48	0.022
Wing fractures ^4^		2	6	0.164	7	1	0.060
Leg fractures	0	0	0	-	0	0	-
DOA	0	0	0	-	0	0	-

^1^ Age 33 days, mean estimated live weight 2000 g; ^2^ Age 44 days, mean estimated live weight 2300 g; ^3^ In seconds per transport module; ^4^ Total number of birds with fractures.

**Table 2:** Comparison of catching methods, broken down to flock.

	LEGS			UPRIGHT		
Indicators	Conventional Hybrid ^1^	Slower Growing Hybrid ^2^	*p*-Value	Conventional Hybrid ^1^	Slower Growing Hybrid ^2^	*p*-Value
Mean crating time ^3^	242.8	261.1	0.053	202.3	260.0	<0.0001
Mean number of birds per drawer	32.2	30.3	0.0001	31.1	29.9	0.0124
Wing fractures ^4^	2	5	<0.001	0	1	<0.001

^1^ Age 33 days, mean estimated live weight 2000 g; ^2^ Age 44 days, mean estimated live weight 2300 g; ^3^ In seconds per transport module; ^4^ Total number of birds with fractures.
